# Feasibility and acceptability of a peer provider delivered substance use screening and brief intervention program for youth in Kenya

**DOI:** 10.1186/s12889-023-17146-w

**Published:** 2023-11-16

**Authors:** Florence Jaguga, Edith Kamaru Kwobah, Ali Giusto, Edith Apondi, Julius Barasa, Mercy Korir, Wilter Rono, Gilliane Kosgei, Eve Puffer, Mary Ott

**Affiliations:** 1 Moi Teaching & Referral Hospital Department of Mental Health, PO BOX 3-30100, Eldoret, Kenya; 2 Columbia University Medical Center/New York State Psychiatric Institute, New York City, NY USA; 3 Moi Teaching & Referral Hospital Department of Child Health and Pediatrics, PO BOX 3-30100, Eldoret, Kenya; 4 Academic Model Providing Access to Healthcare, Eldoret, Kenya; 5 Department of Psychology & Neuroscience, Duke Global Health Institute, Duke University, Durham, NC USA; 6 Division of Adolescent Medicine, Department of Pediatrics, Indiana University, Indianapolis, IN USA

**Keywords:** Peer provider, Substance use, Screening, Brief intervention, Youth, Kenya

## Abstract

**Background:**

Youth in sub-Saharan Africa are at high risk of substance use yet lack access to substance use interventions. The goal of this project was to evaluate the feasibility and acceptability of a peer-delivered, single-session substance use screening and brief intervention program for youth in Kenya.

**Methods:**

This was a convergent parallel mixed methods study utilizing both quantitative and qualitative approaches. Two trained peer providers administered the screening and brief intervention program to 100 youth aged 15–24 years. To evaluate the implementation of the intervention, we collected quantitative and qualitative data. Feasibility and acceptability were quantitatively assessed using the Dissemination and Implementation Measures. Fidelity was assessed by rating all 100 audio-recorded sessions using a checklist. To obtain qualitative feedback on the intervention, we conducted five focus group discussions with 25 youths and six semi-structured interviews with two peer providers and four clinic leaders. The semi-structured interviews were guided by the Consolidated Framework for Implementation Research. Quantitative data was analyzed via descriptive statistics using STATA. Qualitative data was analyzed using thematic analysis with NVIVO.

**Results:**

The lifetime prevalence of any substance use was 50%. The mean level of acceptability of the intervention from the perspective of the youth was 3.53 (SD 0.15), meaning that the youth found the intervention to be acceptable “a lot” of the time. Mean levels of implementation outcomes (acceptability, adoption, Acceptability, Appropriateness, Feasibility, Reach/access, Organizational climate, General leadership skills, and Sustainability) as rated by peer providers and clinic staff ranged between 2.61 (“a moderate amount”) and 4.0 (“a lot”). In qualitative data, youth reported that the intervention was helpful and useful in enabling them to stop or reduce substance use. The peer providers felt that the intervention was easy to implement, while the clinic leaders felt that available resources were adequate, and that the intervention aligned well with the goals of the clinic.

**Conclusion:**

Our findings suggest that the peer-delivered screening and brief intervention program was perceived as acceptable to the youth and feasible to implement.

**Trial registration:**

NCT04998045 Registration date: 10/08/2021.

**Supplementary Information:**

The online version contains supplementary material available at 10.1186/s12889-023-17146-w.

## Background

Globally, youth are disproportionately affected by substance use [1]. Illicit substance use for example remains concentrated among the youth, with peak levels of consumption seen between the ages of 18–25 years of [1]. Youth in sub-Saharan Africa have not been spared. In a systematic review and meta-analysis conducted by Olawole and colleagues [2], the authors found that 41.6% of youth had used at least one substance in their lifetime. In Kenya, the burden of substance use is similarly worrying. A survey conducted among secondary school students across the country found that the lifetime prevalence of substance use was as follows: alcohol (23.4%), khat (17.0%), prescription drugs (16.1%), tobacco (14.5%), and cannabis (7.5%). In that study, the age of initiating the different substances was 13 to 15 years. In a survey conducted among college students in Kenya, the prevalence of any substance use was 69.8% [3].

Young people who use substances often face a myriad of challenges including mental health problems like depression and anxiety [4]; poor performance in school [3] poor relationships with family and friends; accidental injuries; and involvement in crime and offenses [3]. Even worse is that adolescent substance use is associated with faster progression to dependence [5]. For example, in the US, 15.2% of people who start drinking by age 14 eventually develop alcohol abuse or dependence (as compared to just 2.1% of those who initiate use after the age of 21 years) [6]. Addressing substance use among youth is therefore of high priority.

Unfortunately, substance use treatment services in sub-Saharan Africa are generally scarce [7–9] and fragmented [10]. Moreover, youth often lack money to pay for treatment and are often unaware of the available services [10]. In Kenya, treatment for substance use is mainly offered by a few privately owned residential or in-patient facilities [8]. Services are therefore costly and scarce and cannot be accessed by young people.

The World Health Organization (WHO) recommends screening and brief intervention delivered in primary healthcare as a cost-effective population-level intervention for identification and early intervention for risky substance use [11]. In addition, screening and brief intervention is considered best practice by the United Nations Office on Drugs and Crime (UNODC) in the prevention of substance use among adolescents [12]. Screening and brief intervention is generally comprised of 2 parts: (i) Screening, which identifies substance use along a continuum, from no use to high risk, using questions from a validated screening tool; (ii) A brief intervention, which is a short (15–20 min) discussion between a healthcare worker and the patient using motivational interviewing techniques. The goal of the brief intervention is to encourage those with moderate-risk use to reduce or stop substance use to prevent health-related consequences of harmful use, and those with high-risk use to engage with care [11]. Screening and brief intervention delivered by primary healthcare workers has been shown to be effective in reducing low, moderate, and high-risk substance use among adolescents in the US [13, 14], Czech Republic [13], and in South Africa [15].

While screening and brief intervention was originally designed to be delivered by primary healthcare workers, the intervention could be delivered by lay providers, especially in low-to-middle-income countries (LMIC) where primary healthcare workers are few and often face heavy workloads [16]. Peers are lay providers who are age mates or near-age mates with the youth, are about the age of 18–26 years, and are found in many youth and adolescent clinics in Kenya [17, 18] They present a potential means through which screening and brief intervention may be delivered. Peer providers are well placed to deliver screening and brief intervention because they already have basic training in counseling skills. Additionally, they are age-mates with the youth and, therefore can easily relate with them.

Few studies have evaluated the implementation of peer-delivered screening and brief intervention for youth. Winn et al. [19] found that it was feasible to deliver substance use screening and brief intervention for adolescents in the US using trained peer-providers aged 18–28 years. Musyoka et al., [20] explored the perceptions of young peer providers in Kenya, on the feasibility and acceptability of a mHealth-delivered substance use screening and brief intervention program. The authors found that the intervention was feasible and acceptable [20] Our study extends this prior work by obtaining the perceptions of youth, peer providers, and clinic leaders on the feasibility and acceptability of a peer-delivered screening and brief intervention program for youth in Kenya. This project aligns with the Kenyan Ministry of Health guidelines for the delivery of adolescent-friendly services [21] which lists substance use counseling as an essential service, and one target of the Sustainable Development Goals which requires that governments strengthen treatment and prevention of substance abuse.

## Methods

### Study design

This was a mixed-method convergent-parallel study. Both quantitative and qualitative data were separately collected, analyzed, and presented in the results section. The qualitative and quantitative data have been merged in the discussion section and organized by two main themes i.e., perceptions of youth on the intervention and perceptions of staff and peer providers on the intervention.

The evaluation used a single-arm, open-trial design.

### Study setting

The pilot study was conducted at a youth clinic (Rafiki clinic) run by the Academic Model Providing Access to Health Care (AMPATH) [22]. AMPATH is a large chronic disease program in western Kenya and is a partnership between Moi Teaching and Referral Hospital, North American Universities, and the Kenyan Ministry of Health [22].

Rafiki clinic has a total enrolment of 799 youth. Of these, 80% are living with HIV and 99% are aged 15–24 years. Four peer providers work full-time at Rafiki. The peer providers are selected to work at Rafiki based on age (they should be aged 18–24 years), HIV status (must be HIV positive, virally suppressed, and ready to disclose HIV status), and willingness to support youth wellness. Experience with substance use is not considered when hiring peers at Rafiki clinic.

Before the pilot study, all peer providers had received a 5-day training (about 30 h) in HIV adherence counseling, and basic counseling techniques, but none on substance use screening and brief intervention. The training was facilitated by the Pediatrician, Clinical Officers, Psychologists, and Nursing Staff stationed at Rafiki Clinic.

At each clinic visit, the youth first consult with a peer provider in a private room before proceeding for clinician review. During the consultation, peer providers perform antiretroviral therapy adherence counseling, and or offer basic counseling on mental health-related issues that the youth may have such as dealing with stressful situations at school or home. The clinic sees about 300 youth monthly and is staffed by 10 staff directly involved in patient care: 1 pediatrician, 2 clinical officers, 1 psychological counselor, 1 nutritionist, 2 social workers, 1 pharmacist, and 2 nurses.

Rafiki Clinic was set up to address the unique health-related needs of youth and adolescents. For a long time, staff at Rafiki encountered youth with substance use challenges but could not address this problem, hence the need to implement this intervention at the clinic.

### Screening and brief intervention program

The Screening and Brief Intervention program comprised of (i) screening using the ASSIST-Y (Alcohol Smoking and Substance Involvement Screening Test—Youth) questionnaire [23]; and (ii) a single session brief intervention.

We used the ASSIST-Y questionnaire to screen for the level of substance use involvement for all substance types including tobacco products, alcohol, cannabis, cocaine, amphetamine-type stimulants, sedatives, hallucinogens, inhalants, opioids, and 'other' drugs [23].

The first question of the ASSIST-Y asks about lifetime substance use for each of the substances listed above. Endorsement of lifetime use is then followed by an assessment of substance use in the past 3 months. The level of substance involvement is categorized as moderate or high risk and cut-off scores vary for each substance. Scores corresponding to moderate risk substance use are as follows: tobacco products [2-11], alcohol [5-17], cannabis [2-11], cocaine [2-8], amphetamine-type stimulants (ATS) [2-8], sedatives [2-6], hallucinogens [2-8], inhalants [2-8], opioids [2-6] and ‘other’ drugs [2-6]. Scores corresponding to high-risk substance use are as follows: tobacco products [12 +], alcohol [18 +], cannabis [12 +], cocaine [9 +], amphetamine-type stimulants (ATS) [9 +], sedatives [7 +], hallucinogens [9 +], inhalants [9 +], opioids [7 +] and ‘other’ drugs [7 +].

Following the screening, we administered a brief intervention that included either a 5–10-min session of positive reinforcement delivered to youth with no history of substance use over the past 3 months, or a 20–30-min brief motivational interviewing session that was delivered to youth with moderate and high-risk substance use.

Positive reinforcement consisted of personalized feedback on the ASSIST-Y scores, a statement that praised the youth for not using substances, and advice to keep away from substance use in the future. The youth were further given booklets with information on the harmful effects of substances to take home with them.

The brief motivational interviewing intervention was adapted from the WHO ASSIST-linked brief intervention manual. This treatment is based on the FRAMES model (i.e., providing feedback on screening results; ensuring responsibility on the part of the youth; giving clear advice to stop/cut down substance use; giving a menu of options on alternative healthy behaviors to engage in; expressing empathy; and encouraging self-efficacy); and motivational interviewing techniques (creating discrepancy and ambivalence, using open-ended questions, rolling with resistance, reflective listening and summarizing) [11].

The brief motivational interviewing intervention was delivered in 11 steps over a single session as follows: 1. Asking clients if they are interested in seeing their ASSIST-Y scores; 2. Providing personalized feedback to clients about their ASSIST-Y scores; 3. Giving clients advice about how to reduce risk associated with substance use; 4. Allowing clients to take ultimate responsibility for their choices; 5. Asking clients how concerned they are by their ASSIST-Y scores; 6. Weighing up the good things about using the substance against the; 7. less good things about using the substance; 8. Summarizing and reflecting on clients' statements about their substance use with emphasis on the 'less good things'; 9. Asking clients how concerned they are by the 'less good things'; 10. An assessment of readiness or confidence to initiate change using the readiness steps; 11. Giving clients take-home materials to bolster the brief intervention. Youth with high-risk use received a referral to specialist care at the MTRH, Child Psychiatry Out-patient clinic in addition to the brief motivational interviewing [11].

### Adapting the screening and brief intervention program

Before implementation, we adapted the ASSIST-Y and the World Health Organization (WHO) ASSIST-linked brief intervention using the ADAPT-ITT framework [24]. The framework is made up of 8 steps including Assessment, Decision-making, Adaptation, Production, Topical Experts, Integration, Training, and Testing of the evidence-based intervention. The framework has been utilized successfully in adapting a mental health intervention for youth in sub-Saharan Africa [25]. We conducted adaptations to the ASSIST-Y and the WHO ASSIST-linked brief intervention to contextualize them to the Kenyan context and for peer delivery. The adaptations were largely surface-level and comprised of simplifying the language to make it more understandable to the youth, adding instructions to make the manual easy to navigate for the peer providers, and adding street names for the substances to the ASSIST-Y. We maintained the core components of the intervention. Details of the adaptation process have been published elsewhere [26].

### Peer training

In December 2021, we invited all four peer providers who work full time at Rafiki clinic to a training on how to deliver the screening and brief intervention program. Out of these, three completed the training. The training was conducted over 5 days using lectures, quizzes, and role-plays. On each of the days, the training was conducted between 8.00 a.m. and 4.00 p.m. with a 30-min tea break and a one-hour lunch break. We therefore allocated 6 h 30 min for the training on each day (total of 32 h 30 min over the 5-day period). During the training the facilitators delivered lectures that focused on the following areas: Introduction to substance use (types of substances, burden of substance use among youth), myths related to substance use, rationale for screening, screening using the ASSIST-Y, counseling skills, motivational interviewing principles, positive reinforcement, and brief motivational interviewing. The rest of the time was spent on role-plays that helped the peer providers to practice the screening and brief intervention, and counseling skills, with both hypothetical cases and real youth. Details of the training sessions, including content and time allocations have been provided in Supplementary file 1. The training was facilitated by psychologists and psychiatrists on the research team including E.K., W.R., F.J., J.B., G.A., and M.K.

At the end of the training, we conducted exams using standardized role-plays. Each peer provider was examined using 5 standardized role-plays and one real case (a young person with substance use) (Supplementary file 1). To assess competence, three facilitators rated the exam role-plays for each peer, using a fidelity checklist of the main elements of the interventions (see Sect. 2.7.1 of this manuscript for a detailed description of the fidelity checklist). Average scores were obtained for each peer.

Two of the three peer providers achieved satisfactory competence based on assessments by the facilitators during the training. The average fidelity scores were 90%, 86%, and 48% for peer providers 1, 2, and 3 respectively. Peer-providers 1 and 2 were consented and recruited into the study.

### Study participants, recruitment, and study procedures

#### Youth

We recruited 100 youth aged 15–24 years between January and February 2022. We excluded youth who were ill during the appointment, those unable to speak fluently in English, and those who declined to assent or consent. The sample size of 100 was arrived at based on the number of youths seen at the clinic and budgetary considerations. Moreover, the sample size of 100 was large enough to inform us about the feasibility and acceptability of the screening and brief intervention program.

Of the 110 youth who were eligible to participate, ten youths, i.e., four females and six males, declined to participate. Eight out of the nine youth declining to participate were above the age of 18 years. The reasons for declining included: being in a hurry to leave and therefore not having enough time to participate (*n* = 5); declining without any explanation (*n* = 2); and not being comfortable with the content of the study (*n* = 3).

Before data collection, we piloted the study procedures and data collection tools and made adjustments as necessary. A trained research assistant approached all youth presenting for any form of service at the clinic and confirmed eligibility. The research assistant then explained the study procedures and sought assent or consent in English. Consenting or assenting was done in a private room within the clinic. The research assistant collected socio-demographic data from the assenting or consenting youth. The youth then completed quantitative measures of depression (Patient Health Questionnaire-9) and generalized anxiety disorder (Generalized Anxiety Disorder -7). Thereafter the peer providers administered the screening and brief intervention program to youth with moderate and high-risk substance use. Youths with high-risk substance use were additionally referred for specialist mental health treatment. Youth who had no history of lifetime substance use or those who had not used any substance in the past 3 months received verbal positive reinforcement, brief advice on the harmful consequences of substance use, and booklets with content on the harms of substance use.

One peer-provider delivered the intervention to 52 youth, and the other to 48 youth. The youth were conveniently assigned to the two peer providers. This is because the goal was to explore feasibility, so the intervention was integrated into routine clinic procedures and participants were assigned to peer providers based on whomever was available to deliver the intervention to the youth.

Immediately after the screening and brief intervention program, each youth completed quantitative measures of intervention acceptability. Overall, of the 100 youth screened, 63 received positive reinforcement, 35 received brief motivational interviewing, and 15 received brief motivational interviewing with referral to specialized treatment. Two youths who were to receive the brief motivational interviewing declined to see their ASSIST-Y scores and to continue with the brief motivational intervention. They were thanked for their time and given the substance use education booklets. Each youth was compensated USD 5.00 for the time they spent at the clinic.

Youth with moderate to severe depression and anxiety were referred to the Psychologist within Rafiki Clinic for further assessment.

We held five supervision sessions during the screening and brief intervention implementation period. The sessions were facilitated by two psychologists i.e., W.R., and J.B. During supervision, the peer providers gave feedback on the sessions, and challenging areas were addressed through role-plays. Fidelity to the screening and brief intervention program was assessed by audio-recording all the sessions and rating them using a checklist of key elements of the intervention. The peer providers were reimbursed USD 5.00 for every participant recruited.

#### Peer providers

We recruited two peer providers who had achieved competency at the peer provider training into the study. We obtained informed consent from the peer providers before data collection began.

#### Clinic leaders

We purposively identified four clinic staff who were involved in major decision-making at the clinic and recruited them into the study. We obtained informed consent from the clinic leaders before data collection began.

#### Implementation of the peer-delivered screening and brief intervention and peer provider supervision

The peers implemented the screening and brief intervention program between January and February 2022 for the recruited youth.

### Quantitative data collection and analysis

#### Quantitative* data collection*

Researcher-designed questionnaires were used to collect socio-demographic data from the study participants. Youth socio-demographic data included: age, sex, marital status, living arrangement, level of education, and parental status. Peer provider socio-demographic data included: age, gender, level of education, and duration of work at Rafiki clinic in years. Clinic leaders’ socio-demographic data included: age, gender, cadre, highest level of education, and duration worked at Rafiki clinic in years.

The Patient Health Questionnaire-9 (PHQ-9) was used to collect data on depression. The PHQ-9 is a valid and reliable tool for measuring the severity of major depression. It is a 9-item tool that examines symptoms over the past two-week period. Each of the 9 items is rated as follows: 0 – “not at all”, 1 – “Several days”, 2 – “More than half the days”, 3 – “Nearly every day”. In a study conducted among Kenyan adolescents, the PHQ-9 was found to be a reliable measure of depression (Cronbach’s alpha 0.73) [27]. In that study, the cut-offs determined by Kroenke 2001 [28] were used i.e., 0–4 minimal depression, 5–9 mild depression, 10–14 moderate depression, 15–19 moderately severe depression, and 20–27 severe depression.

We measured levels of Generalized Anxiety Disorder using the Generalized Anxiety Disorder -7 (GAD-7) scale. It is a valid and reliable tool for measuring the severity of GAD. It is a 7-item tool that examines symptoms over the past two-week period. Osborn et al. [27] examined the psychometric properties of GAD-7 among Kenyan adolescents and reported that the reliability was adequate (Cronbach's alpha for the present study was 0.78). GAD-7 has been used to evaluate anxiety among Kenyan youth [27]. In that study, cut-offs determined by Kroenke et al. were used i.e., mild anxiety [5–9], moderate range [10–14], and severe range [15–21, 29].

We conducted surface adaptations on the PHQ-9 and GAD-7 (e.g., simplifying the language) to make the items more understandable to the youth.

We obtained youth feedback on the acceptability of the screening and brief intervention program using the Dissemination and Implementation Measures—Consumer tool, acceptability module. The tool assesses intervention acceptability from the perspective of the recipient of the intervention. The module has 15 questions rated on a 4-point scale as follows: 1- Not at all; 2- a little bit; 3- a moderate amount; 4- a lot.

We obtained peer provider and clinic leaders’ feedback on the screening and brief intervention program using ‘Dissemination and Implementation Measures’ – provider and organization tools respectively [30]. Each tool is comprised of the following modules: Adoption, Acceptability, Appropriateness, Feasibility, Reach/access, Organizational climate, and General leadership skills. The organization tool has an additional module on Sustainability. Each module comprises of 12–15 questions rated on a 4-point scale as follows: 1- Not at all; 2- a little bit; 3- a moderate amount; 4- a lot [30].

The Dissemination and Implementation Measures were developed for LMICs by researchers from John Hopkins University [30]. The tools were developed based on the implementation science outcomes of Adoption, Acceptability, Appropriateness, Feasibility, and Reach/Penetration by Proctor and colleagues [31]. We conducted surface adaptations (e.g., simplifying the language) on the tool as allowed by the developers to tailor it to our context.

Fidelity ratings of the audio-recorded session (all 100 of them) were conducted using a checklist of key elements of the screening and brief intervention program. The fidelity tool was divided into four sections corresponding to the different parts of the intervention: Screening (2 items with a maximum score of 4); positive reinforcement (6 items with a maximum score of 12); brief motivational interviewing (17 items with a maximum score 34); referral to treatment (1 item with a maximum score of 2). Each item was rated on a 3-point scale as follows: Not at all i.e., the peer did not do the step at all (0); Partially completed i.e., the peer tried but did not complete it or did not do it well) (1); The peer completed the step and did it well (2). Before rating, two members of the research team i.e., F.J. and J.B. each rated the first 10 sessions and achieved 95% agreement before moving on to independent rating.

### Quantitative data analysis

Descriptive statistics were used to summarize the socio-demographic, substance use, and mental health characteristics of youth. Levels of the various implementation outcomes were obtained by calculating mean scores across all items of the fidelity, and dissemination, and implementation measures.

### Qualitative data collection and analysis

#### Youth focus group discussions

Between 8^th^ and 31^st^ March 2022, we conducted five focus group discussions (FGD) with 25 youth to explore their perceptions on the acceptability of the screening and brief intervention program. The youth were purposively identified, and FGDs were conducted, based on age, gender, and substance use risk, to ensure that the youth were comfortable enough to express their opinions. Supplementary file 2 provides details on the composition of the FGDs. The FGDs were conducted in a meeting room at a tertiary-level health facility in Eldoret. The FGD guides explored areas such as youths' perceptions of the session content, perceptions about their interaction with the peer providers, and recommendations for improvement. The FGD sessions were led by experienced moderators M.K., W.R., and J.B. and were conducted in English. The FGDs started with the consenting process followed by an introductory session to ensure a comfortable and relaxed atmosphere. The discussions lasted an average of one hour and twenty-two minutes and were audio-recorded. Participants were reimbursed USD 5.00 for their time.

#### Peer-provider and clinic leaders semi-structured interviews

In April 2022, we conducted six individual semi-structured interviews with two peer providers and four clinic leaders (psychologist, nurse, pediatrician, and clinical officer) to explore their perceptions on the feasibility and acceptability of the screening and brief intervention program. The interviews were conducted at a private place within the Rafiki clinic. The semi-structured interview guides were developed based on the Consolidated Framework for Implementation Research (CFIR) framework. The CFIR lists a comprehensive set of implementation determinants organized into 5 domains: intervention characteristics, outer setting, inner setting, characteristics of individuals, and executing [32] For the peer provider interview guides, we included questions that explored all five domains of the CFIR i.e., intervention characteristics, outer setting, inner setting, characteristics of individuals, and executing (Supplementary file 3). For the clinic leaders’ interview guide, we included questions that explored four CFIR domains including outer setting, inner setting, characteristics of individuals, and executing (Supplementary file 3).

The interviews were led by experienced moderators M.K., W.R., and J.B., and were conducted in English. The interviews started with the consenting process followed by an introductory session. The semi-structured interviews lasted an average of 42.3 min and were audio-recorded. Participants were reimbursed USD 5.00 for their time.

#### Qualitative data analysis

##### Youth FGDs

The audio-recorded interviews were transcribed verbatim by G.K. and then entered into NVivo for analysis. The transcripts were reviewed, and initial coding was done separately by F.J. and M.K. using an inductive approach. The two discussed the codes and sub-codes and resolved initial disagreements to develop a refined codebook. The final coding of the transcripts was done by F.J. and M.K. using the refined codebook.

F.J., M.K., and M.O. then performed thematic analysis to identify codes that fitted into themes that addressed the question of whether the screening and brief intervention program was acceptable to the youth. The themes were developed and defined through a process of discussion amongst the three authors (F.J., M.K., and M.O.) until a consensus was arrived at. The codes and sub-codes fit into five themes i.e., youths' perceptions of the screening and brief intervention program content and delivery; youths' perceptions of the peer providers; the impact of the intervention on youth behavior; youths' perceptions of usefulness of the intervention; and recommendations for improving the screening and brief intervention program.

##### Peer-provider and clinic leadership semi-structured interviews

The audio-recorded interviews were transcribed verbatim by G.K. and then entered into NVivo for analysis. Initial coding was done by F.J. and M.K. guided by the CFIR domains and constructs. The codes and sub-codes were iteratively reviewed and discussed by F.J. and M.K. to ensure that the coding of the domains was being done as intended. A final codebook was developed, and final coding was done by F.J. and M.K. independently.

**Fig. 1:**
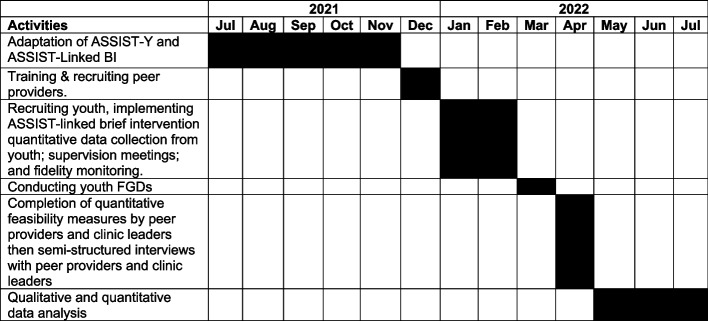
Timeline of study activities

Figure 1 below provides a timeline of the study activities for this project.

## Results

### Participant characteristics

#### Youth socio-demographics

A total of 100 youth consented to participate in the study. The mean age for the participants was 17.33 years (SD 1.53). The slight majority were male (59%), had never married (98%), were living with a family member or a relative (89%), and were HIV positive (81%). Table 1 shows their socio-demographic characteristics.

**Table 1:** Youth socio-demographic characteristics (*n* = 100)

Socio-demographic characteristic	Mean (SD)/ Frequency (%)
Age (yrs)	
Mean (SD)	17.33 (1.53)
Range	16.00—19.00
Gender	
Male	59 (59.0%)
Female	41 (41.0%)
Other	0 (0.0%)
Education level	
No Primary	0 (0.0%)
Incomplete primary	2 (2.0%)
Complete primary	1 (1.0%)
Incomplete Secondary	46 (46.0%)
Complete secondary	19 (19.0%)
Tertiary +	32 (32.0%)
Marital status	
Never married	98 (98.0%)
Separated/divorced/widowed	0 (0.0%)
Married/cohabiting	2 (2.0%)
Living arrangement	
Family/relative	89 (89.0%)
friend/non-relative	2 (2.0%)
Alone	9 (9.0%)
Parental status	
Both parents alive	44 (44.0%)
One parent alive	36 (36.0%)
Both parents died	20 (20.0%)
HIV status	
Positive	81 (81.0%)
Negative	8 (8.0%)
Unknown	11 (11.0%)

#### Youth mental health characteristics

The mean PHQ-9 score was 4.14 (SD 3.74). Eighty-six percent of the participants had some level of depression (a score of 1 and above on the PHQ-9); The mean GAD-7 score was 3.16 (SD 3.58). Seventy-two (72%) youth had some level of GAD symptoms (a score of 1 and above on the GAD-7) (Table 2).

**Table 2:** Mental health characteristics of the youth (*n* = 100)

	**Mean (SD)/ Frequency (%)**
**PHQ-9 scores**	
Mean	4.14 (SD 3.74)
Range	1–27
**PHQ-9 severity (PHQ-9 scores)**	
No depression (0)	14 (14.0%)
Minimal depression (1–4)	49 (49.0%)
Mild depression (5–9)	26 (26.0%)
Moderate depression (10–14)	8 (8.0%)
Moderately severe depression (15–19)	3 (3.0%)
Severe depression (20–27)	0 (0.0%)
**Level of difficulty with functioning for those with PHQ-9 score of 1 and above**	
Not difficult	29 (29.0%)
Somewhat difficult	41 (41.0%)
Very difficult	11 (11.0%)
Extremely difficult	5 (5.0%)
Not applicable (N/A)	14 (14.0%)
**GAD-7 scores**	
Mean (SD)	3.16 (SD 3.58)
Range	1–21
**GAD-7 severity**	
No anxiety (0)	28 (28.0%)
Minimal anxiety (1–4)	45 (45.0%)
Mild anxiety (5–9)	20 (20.0%)
Moderate anxiety (10–14)	6 (6.0%)
Severe anxiety (15–21)	1 (1.0%)
**Level of difficulty with functioning for those with a GAD-7 score of 1 and above**	
Not difficult	23 (23.0%)
Somewhat difficult	32 (32.0%)
Very difficult	13 (13.0%)
Extremely difficult	4 (4.0%)
Not applicable (N/A)	28 (28.0%)

#### Youth lifetime substance use

The lifetime prevalence of any substance use was 50%. The lifetime prevalence was highest for alcohol (42%) followed by cannabis (16%), khat (14%), and tobacco (10%). None (0%) of the youth reported inhalant, opioid, or injecting drug use (Table 3).

**Table 3:** Lifetime substance use (*n* = 100)

Substance	Freq (%)
Tobacco	10 (10.0%)
Alcohol	42 (42.0%)
Cannabis	16 (16.0%)
Cocaine	2 (2.0%)
Khat	14 (14.0%)
Sedative	1 (1.0%)
Hallucinogen	1 (1.0%)

#### Youth mean ASSIST-Y scores

The mean ASSIST-Y scores were highest for alcohol at 6.38 followed by cannabis (5.65) and tobacco (2.63). These scores all correspond to moderate risk use (Table 4). None of the youth reported the use of hallucinogens, opioids, or inhalants in the 3 months before the interview. Additional youth substance use characteristics have been provided in Supplementary file 4.

**Table 4:** Youth mean ASSIST-Y scores

Substance	Mean (SD) ASSIST Score
Tobacco score	2.626 (0.43)
Alcohol score	6.382 (3.55)
Cannabis score	5.651 (1.64)
Cocaine score	0.300 (0.03)
Khat score	4.025 (1.11)
Sedative score	2.549 (0.36)

#### Sociodemographic characteristics of the peer providers

Two peer providers (one male and one female) participated in the study. The mean age for the peers was 26 years. Both had attained tertiary-level education. Their mean duration of work at the Rafiki clinic was 2 years.

#### Socio-demographic characteristics of the Rafiki clinic leaders

A total of four clinic leaders participated in the study. All the leaders were female. They had a mean age of 42 years. The cadres of the clinic leaders were as follows: one pediatrician, one psychological counselor, one clinical officer, and one nurse. They had worked for a mean of 5.75 years (SD 3.30 years; range 2.0–10.0 years) at Rafiki clinic. All the clinic leaders had attained tertiary-level education.

### Quantitative feedback on the screening and brief intervention program

#### Quantitative ratings of acceptability from the perspective of the youth

The overall mean score for acceptability was 3.53 (SD 0.15) corresponding to “a moderate amount” (each question was rated on a 4-point scale as follows: 1- not at all; 2- a little bit; 3- a moderate amount; 4- a lot). The item scoring lowest was “Did you feel that the peer addressed any questions or concerns you had about the screening and brief intervention program?” at 3.09 (Table 5).

**Table 5:** Acceptability ratings from the perspective of the youth (*n* = 100)

Item	Mean score^a^ (SD)
**The overall mean score for acceptability**	**3.53 (0.15)**
Overall, did you like the screening and brief intervention program?	3.35 (0.98)
Did you like attending the screening and brief intervention program sessions?	3.59 (0.80)
Did you feel satisfied with the screening and brief intervention program services received?	3.42 (0.97)
Did you enjoy learning about screening and brief intervention programs?	3.71 (0.67)
Do you feel that the skills you learned in the screening and brief intervention program are useful?	3.57 (0.82)
Do you feel that the components of the screening and brief intervention program make sense to you?	3.72 (0.78)
Did you feel comfortable raising questions to your peer-provider?	3.63 (0.68)
Did you feel that the peer provider listened to your concerns and questions about the screening and brief intervention program?	3.52 (0.96)
Did you feel satisfied with your peer provider's abilities during the screening and brief intervention program?	3.49 (0.92)
Did you feel that your peer provider addressed any questions or concerns you had about the screening and brief intervention program?	3.09 (1.13)
Did your peer-provider take an interest in you?	3.64 (0.79)
Did you feel that you could trust your peer-provider?	3.48 (0.97)
Did you feel that you understood the way things were explained to you during the intervention?	3.72 (0.59)

^a^ Each question was rated on a 4-point scale as follows: 1- Not at all; 2- a little bit; 3- a moderate amount; 4- a lot

#### Quantitative ratings of screening and brief intervention program by the peer providers and clinic leaders

The implementation outcomes that had the highest mean ratings from the perspective of peer providers were 'general leadership skills' at 4.00 (SD 0) and 'acceptability' at 3.96 (SD 0.21) (each question was rated on a 4-point scale as follows: 1- not at all; 2- a little bit; 3- a moderate amount; 4- a lot). The implementation outcome that had the highest mean rating from the perspective of the clinic leaders was ‘appropriateness’ at 3.79 (SD 0.28). The lowest-scoring implementation outcome from the perspective of the clinic leaders was feasibility at 2.83 (SD 0.81) (Table 6).

**Table 6:** Ratings of implementation outcomes from the perspective of the peer providers

	**Mean score (SD)**^a^	
**Implementation outcome**	**Peer-providers (*n* = 2)**	**Clinic leaders (*n* = 4)**
Adoption	3.11 (0.97)	3.66 (0.5)
Acceptability	3.96 (0.14)	3.75 (0.25)
Appropriateness	3.90 (0.21)	3.79 (0.28)
Feasibility	3.82 (0.37)	2.83 (0.81)
Reach/access	3.69 (0.46)	2.61 (0.38)
Organizational climate	3.91 (0.26)	3.55 (0.54)
General leadership skills	4.00 (0)	3.72 (0.49)
Sustainability	-^b^	3.25 (0.96)

^a^Each question was rated on a 4-point scale as follows: 1- not at all; 2- a little bit; 3- a moderate amount; 4- a lot

^b^The provider tool does not have a section on sustainability

#### Fidelity ratings for the screening and brief intervention

Adherence to the steps in the different parts of the screening and brief intervention program was highly rated at 97.0% for screening, 97.3% for positive reinforcement, 91.4% for brief motivational interviewing, and 93.5% for referral to treatment. The complete findings of the fidelity assessments have been provided in Table 7 below.

**Table 7:** Fidelity ratings for the screening and brief intervention program

Parts of the screening and brief intervention program	Mean scores (SD)	Minimum and maximum scores for each section	Percentage
Screening (*n* = 100)	3.88 (0.38)	0–4	97.0%
Positive reinforcement (*n* = 63)	11.68 (1.35)	0–12	97.3%
Brief motivational interviewing (*n* = 35^a^)	31.09 (2.99)	0–34	91.4%
Referral to treatment (*n* = 15)	1.87 (0.52)	0–2	93.5%

^a^This number is not 37 as expected because 2 youths with moderate/high risk declined to see their ASSIST-Y scores and to continue with the brief intervention. They were thanked for their time and given the substance use education booklets

#### Time taken to deliver the screening and brief intervention program

The mean time taken for the peer providers to deliver the screening and brief intervention sessions was 12.17 min (SD 8.22 min).

### Qualitative findings

#### Feedback from youth on the screening and brief intervention program

The youth’s perceptions gathered through the focus groups were organized into 5 themes: youths' perceptions of the screening and brief intervention program content and delivery youths' perceptions of the peer providers, the impact of the intervention on youth behavior, youths' perceptions of usefulness of the intervention, and recommendations for improving screening and brief intervention program.

The youth expressed mixed views about the intervention content and delivery. Some youth felt that the content of the session was relatable to them and that the sessions had been conducted privately and confidentially.

*“I felt comfortable because the peers were friendly, and they were so interactive and the questions and discussion they were talking relate to our environment as youth”*

A few youths however felt that the questions asked during the screening and brief intervention program evoked negative emotions. One youth noted: *“There were some questions that were very confrontational…”.*

A recurring theme was that the youth liked the way they communicated with and interacted with the peer providers. They felt that they could trust the peer providers and were comfortable with them. Many youths felt that the peer providers were friendly, open, and skilled enough to deliver the intervention. They felt that the peer providers listened to them well and addressed their concerns. One noted that *“the peer was friendly, so [they] were very comfortable asking questions.”* In addition, they liked the way the peer providers were straightforward and explained things well.

*“For example, on the effects of using alcohol, he informed me that I am going to be broke, of which every Monday I am usually broke because of spending on alcohol over the weekend. So, I was satisfied with the fact that he was telling me the truth.”*

Most youth reported that they felt comfortable with the peer providers. Reasons for feeling comfortable included that the peer providers were age mates with the youth, were friendly, and were of a similar gender as the youth because the peer providers assured them of confidentiality, and because they had shared experiences with the youth.

*"I took him as a brother…the way he approached me and began talking to me and when he began asking me questions, I just trusted him…the way he was talking to me he didn't seem to have bad intentions, so I just trusted him".*

A few youths however felt uncomfortable with the peer providers either because they had met them before, were unfamiliar with them, or they felt that the peer providers were too serious and judgmental.

*“For me it was good but there were some personal problems that if we haven't met with the peer several times, it will be so hard to communicate"*

A few youths felt that the peer providers were ill-prepared for the session. They recommended that the peer providers read and understand the intervention before the actual session. One youth noted *" … I felt like he/she should go through the questions first so I should not feel he/she is just reading it, I should feel they own it”.*

A few youths also felt that the peer providers should do more research so that they improve their knowledge and are better able to answer the youths’ questions. One youth said that *"[the peers should] do more research to get more information [so that they can better answer young people’s questions]”.*

Many youths who were using substances gained insight into their harmful patterns of substance use and had already taken steps towards quitting or cutting down on substance use following the intervention.

*"I received information about the drugs, I knew how they affect people and I decided to leave it."*

The most mentioned strategy for stopping or reducing substance use that the youth had employed was avoiding friends who use:

*"It taught me not to walk with friends who use substances. Initially, there was no party that I could miss but for now, since we closed schools there have been parties, but I have not attended any. So, the free time I have I'd rather use it to study rather than go to parties."*

A recurring theme was that the youth felt that the screening and brief intervention program content was useful, with information about the harmful effects of substance abuse being reported by most youth as the most useful. Many youths reported that they did not have prior knowledge of the negative effects of substances.

*“The information that was most helpful was the effects of the drugs in the body like the brain.”*

The youth recommended that the intervention be translated to Swahili, or other local languages to ensure that it was accessible to many youths. They further recommended that the length of sessions and number of sessions be increased to allow room for them to give explanations, and so that they receive additional information on the types and effects of substances.

*“Use of other language, Rafiki has diverse individuals, and all do not understand English or Swahili so they can use mother tongue”.*

They further recommended that the peer providers share their experiences on substance use with the youth.

*"The peer to be part of the discussion, for instance, them [the peer provider] saying I was an addict, or I was using something, he/she should not just be projecting the issue to only me…”*

#### Perceptions of peer-providers (*n* = 2) and clinic leadership (*n* = 4) guided by the CFIR

##### Characteristics of individuals

Overall, all peer providers and clinic leaders were confident that Rafiki Clinic would continue to implement the screening and brief intervention program after the study was over.

*Peer provider:“I am very confident. After the training and three months of administering [the screening and brief intervention program], I am very confident…I have grasped a lot of the intervention …, so with or without the [manual] I can administer [the intervention].”*

*Clinic leader:“[I am] really confident [that] we will be able to implement [screening and brief intervention program because] ...we have very supportive staff, …we have space, and…we have a good referral system…”*

Both peer providers reported that their experience delivering the intervention was good.

*“My experience has been good; I have even discovered that I could be a counselor."*

##### Inner setting

All the clinic leaders believed that the intervention was compatible with the overall goal, vision, and mission of Rafiki Clinic. The peer providers and clinic leaders perceived that it was possible to integrate the screening and brief intervention program into existing Rafiki clinic processes and that the screening and brief intervention program would not conflict with other services or processes at the clinic. All clinic leaders reported that Rafiki was open to adopting new ideas and had successfully implemented several new programs in the recent past. All clinic leaders felt that implementing the screening and brief intervention program would be of high priority but that addressing loss to follow-up of youth on antiretroviral therapy, and sexual and reproductive health could be of higher priority. The peer providers believed that the intervention fit in well with the roles and responsibilities of peer providers at Rafiki Clinic.

*Clinic leader: "Our mission and vision is to have a healthy young population because they are our tomorrow and so the adolescent period is the period for risk-taking and exploration and so it is the ideal period for prevention and so having this screening and brief intervention program in the prevention bit works perfectly with our goal but also for the intervention part for those who have already begun [using]”*

*Peer provider"…[the] first person to engage the client is us the peers, so the moment I am tackling different issues while I am doing [the routine] one-on-one session [with the youth] then that is the moment we can [implement the screening and brief intervention program] …”*

The clinic leaders and peer providers all reported that Rafiki frequently saw youth with substance use problems but did not have an established program for addressing those problems. The clinic leaders all felt that the screening and brief intervention program would adequately meet the needs of the youth at Rafiki.

*Clinic leader: “I think one of the strong points in my view is the fact that [the screening and brief intervention program] was being done routinely because most of the time we were not asking that routinely, so it has helped us in terms of talking to youths who are not using it and therefore creating awareness and then hopefully preventing for those who have started or who show high risk for substance use, and of course the fact that it is being delivered by the youth themselves makes it youth friendly which hopefully the uptake should be better by the clients”*

The clinic leaders and peer providers felt that there was adequate staffing and space within the Rafiki clinic to allow for the implementation of screening and brief intervention program. The clinic leaders reported that the peer providers who took part in the study were fully stationed at the clinic and would continue to provide the intervention after the study was over. Concerning funding, some clinic leaders felt that there was no need for additional funding to implement the screening and brief intervention program while others felt that additional funding would be needed to support ongoing training for the peer providers and clinic staff and to provide transport for youth to return for follow-up visits.

Peer provider: “… yeah there is a big space here in Rafiki.”

##### Intervention characteristics

Overall both peer providers reported that the screening and brief intervention program was fairly simple and easy to learn and deliver.

*Peer provider: “…screening was easy to implement…because [I had been trained on it] …I didn't see any difficulty [with the brief intervention either].”*

##### Outer setting

There was limited awareness among the clinic leaders of the national or regional policies that address substance use. Two clinic leaders and one peer provider were aware of existing programs that supported youth substance use (mostly provision of education) within the region but reported that there was limited interaction between these existing youth programs and Rafiki.

*Clinic leader: “…am here we don't go to the other center to find out what they are doing.”*

*Peer provider:* “…*[the other youth program] sees Rafiki as a competition so in most cases we don't interact because they say we [take away] their clients.”*

##### Process

The clinic leaders and peer providers all felt that the implementation of the screening and brief intervention program largely went well. The peer providers felt that the space that had been allocated for the screening and brief intervention program was adequate and that there was enough privacy. Both peer providers also felt that the weekly supervision was useful:

*Peer provider:“[the supervision] was very useful [I received correction for the parts I did not administer well]…it was not like someone was being downgraded but uplifted… so you gain confidence in yourself…[the supervision] made me feel like a counselor.”*

A few challenges noted during implementation by both peer providers included the fact that background noise from the table tennis players and the music was a problem. In addition, the peer providers reported that the youth mentioned certain substance names that the peer providers were unfamiliar with.

Peer provider: "You could find some clients mentioning different [substance] names which I didn't understand…[e.g. cookies and mushroom]”.

The clinic leaders and peer providers felt that it would be important to have champions to advocate for the implementation of the program. They felt that several clinic staff could serve as champions e.g. the nurses, psychologists, peer providers, and nutritionists. The clinic leaders felt that the champions could play a role in supervising the peer providers, advocating for funding, and linking Rafiki to external youth programs.

Peer provider: “… our in charge, doctors, nurses, I think the all family of Rafiki can [be screening and brief intervention program champions].”

##### Recommendations by clinic leaders and peer providers

The clinic leaders recommended that clinic staff be trained on the screening and brief intervention program so that they can understand what it is about. They also recommended that a digital version of screening and brief intervention program for the youth be developed.

One peer-provider recommended that the screening and brief intervention program be delivered in Swahili. Both peer providers highlighted the need to follow up with the youth to check for progress. One peer provider felt that to ensure that the screening and brief intervention program is implemented after the grant, it would be important to make the program mandatory and have regular refresher training for the peer providers.

## Discussion

The goal of this paper was to describe the feasibility and acceptability of a peer-delivered substance use screening and brief intervention program for youth aged 15–24 years.

### Perception of youth on the acceptability of the screening and brief intervention program

The youth found the intervention to be acceptable. Our findings broadly concur with those of other studies that have evaluated the acceptability of substance use brief interventions among youth. In a study conducted by Bonar et al. [33] among youth in the US, the authors found that the rating for overall liking for the substance use brief intervention was 79% and finding it helpful to discuss substances was 71%. Carney et al. [15] qualitatively explored the acceptability of a substance use brief intervention for adolescents in South Africa. In that study, the adolescents reported that they found information on the intervention content and the delivery to be acceptable and that the intervention was able to bring about behavior change.

Brief advice on the harms of substance use is a key component of brief interventions that contribute to their effectiveness. It is therefore important to highlight that many youths in our study found the information on the harms of substance use to be most helpful. Carney et al., [15] explored the acceptability of a substance use brief intervention among adolescents in South Africa. Similar to our study, the adolescents reported that the information on substance use harms was the most valuable. Learning about the harms of substance use can be useful in assisting youth to make informed choices about using substances. Often myths about the effects of substances abound and this can facilitate substance use. Giving youth accurate information is therefore important.

The item that scored lowest in our study was “Did you feel that the peer provider addressed any questions or concerns you had about the screening and brief intervention program?” This low rating is supported by feedback from the youth during the FGD. The youth felt that the peer providers needed to do more research so that they could better answer the youths’ questions. The youth further recommended that the peer providers read and understand the intervention well before the session so that the actual session is more interactive. In a study conducted in Tanzania to explore peer perceptions on delivering an HIV adherence program to youth, it was reported that the peer providers had gaps in knowledge concerning technical topics like viral resistance [34]. This finding highlights the importance of continuous supportive supervision and mentorship for lay providers. This has been emphasized as one of the best practices as regards task-shifting [35, 36].

Despite the low score on the item above, other items relating to youth perceptions concerning the peer-providers were rated between 3.48 and 3.72 indicating an overall, acceptability of peer-provider delivery of the screening and brief intervention program by the youth. These findings were corroborated during the FGDs. The youth reported that they enjoyed their interactions with the peer providers, trusted them, and felt comfortable talking to them. Similar findings have been reported elsewhere. In a study exploring the acceptability of a peer-delivered antiretroviral therapy adherence and substance use intervention among South African youth, the youth reported that peer delivery made it easier to share their experiences and helped them feel heard when doing so [37]. Peer delivery for youth substance use interventions has several potential benefits including the provision of empathic and relatable support to the youth [38], and improvement in retention in care [34], which in turn have the potential to improve intervention outcomes.

An important goal of the screening and brief intervention program is to bring about behavior change. The youth rated the usefulness of skills learned during the program at 3.57 (SD 0.82). During the qualitative interviews, the youth reported that they had utilized skills learned during screening and brief intervention program to reduce or stop their substance use. This finding is similar to that reported in a study exploring the acceptability of a substance use brief intervention for youth in South Africa. In that study, the youth reported that the brief intervention motivated them to change their substance use behaviors [15]. In a study by Maslowsky et al., [39] most youth reported that they intended to quit or reduce substance use following receipt of a screening and brief intervention.

A key recommendation was that the youth wanted more room to explain and share their thoughts and wanted the peer providers to share their substance use experience. Brief interventions by their nature are short and their goal is to motivate behavior change and encourage engagement with therapy. Their depth is therefore often limited, as compared to other detailed therapies such as cognitive behavior therapy. Nonetheless, this recommendation by youth highlights the need to implement longer-term therapies at the Rafiki clinic that provide opportunities for additional or follow-up therapy. Currently, plans to implement a five-session group-based substance use therapy are underway for youth with high-risk use. This finding also suggests that the screening and brief intervention program met its goal of piquing youth interest in getting engaged in substance use treatment.

In addition to the recommendation above, the youth and peer providers proposed that the intervention be delivered in the local language i.e., Swahili. The youth further recommended that the menu of options be revised to make them more practical for the youth. These recommendations will be incorporated in a refined version of the screening and brief intervention program in preparation for a pilot randomized controlled trial.

### Perception of peer providers and clinic leaders on the screening and brief intervention

Quantitative and qualitative feedback from the peer providers and the clinic leaders were largely positive providing additional support for the feasibility of the screening and brief intervention program. The peer providers felt that the intervention was simple and easy to implement. Other studies have similarly found that peer providers can feasibly implement mental health interventions for adolescents and youth [34, 40]. Generally, the clinic leaders felt that Rafiki Clinic had adequate resources and was able to continue implementing the screening and brief intervention program beyond the grant. They also felt that screening and brief intervention program aligned well with the overall goals of the clinic. Our findings concur with those of a study conducted by in the US by Monico et al. [41]. The authors found that the perceptions of organizational staff concerning screening, brief intervention, and referral to treatment for adolescent substance use were largely positive. Support for the screening and brief intervention program from the Rafiki clinic leadership is important because they (clinic leaders) play a significant role in determining the allocation of resources and setting priorities within the clinic.

This study has several limitations. First, it was conducted in a single center so findings may not be generalizable to other youth clinics across Kenya. Secondly, the sample size for the peer providers and clinic staff was small. Nonetheless, our findings provide useful data which may be useful in guiding further research in the field.

Based on the findings of this study, we will refine the screening and brief intervention program manual. Specifically, we will translate it to Swahili as proposed by the youth and refine the menu of options. Further, we will conduct additional training for the peer-providers on the program based on recommendations by the youth. For example, we will offer additional training on types of substances and how to show unconditional positive regard during sessions. We then plan to conduct a pilot randomized control trial of the intervention to explore the feasibility of conducting a full-scale trial.

## Conclusion

The findings of this study show that it is feasible and acceptable to implement a peer-provider-delivered substance use screening and brief intervention program for youth in Kenya. Further research is needed to test the effectiveness and economic viability of this program when implemented on a large scale.

### Supplementary Information

**Additional file 1: Supplementary file 1. **Content for the peer provider training.

**Additional file 2: Supplementary file 2. **Composition of the focus group discussions.

**Additional file 3: Supplementary file 3. **Semi structured interview guides for peer providers and clinic leaders.

**Additional file 4: Supplementary file 4. **Additional data on youth substance use characteristics.

## Data Availability

The datasets used and/or analyzed during the current study are available from the corresponding author on reasonable request.

## Abbreviations

ADAPT-ITT: Assessment, Decision making, Adaptation, Production, Topical Experts, Integration, Training, Testing the evidence-based intervention
AMPATH: Academic Model Providing Access to Healthcare
ASSIST-Y: Alcohol Smoking and Substance Involvement Screening Test—Youth
CFIR: Consolidated Framework for Implementation Research
FGD: Focus Group Discussion
FRAMES: Feedback Responsibility Advice Menu of options Empathy Self-efficacy
GAD-7: Generalized Anxiety Disorder -7
HIV: Human Immunodeficiency Virus
LMIC: Low- and Middle-Income Country
PHQ-9: Patient Health Questionnaire-9
SD: Standard Deviation
US$: United States Dollar
UNODC: United Nations Office on Drugs and Crime
US: United States
WHO: World Health Organization
