# Supplementary material for: Feasibility and acceptability of a peer provider delivered substance use screening and brief intervention program for youth in Kenya
Source: BMC Public Health. 2023 Nov 16;23:2254. doi: 10.1186/s12889-023-17146-w (PMC10652467; doi:10.1186/s12889-023-17146-w)
Supplement: Supplementary file 1 — Additional file 1: Supplementary file 1. Content for the peer provider training. [file 12889_2023_17146_MOESM1_ESM.docx]

**Supplementary file 1: Content for the peer provider training**

|  | **Topics** | **Mode of learning** | **Time allocated** |
| --- | --- | --- | --- |
| **Day 1** | Pre-test | Twenty Multiple Choice Questions | 30 min |
|  | About the study: justification, aims, research procedures, project timelines |  | 30 min |
|  | Introduction to substance use: definition, types of substances; burden of substance use among youth; common questions youth may ask about substance use and how to respond; myths about substance use | Lecture | 1 hour |
|  | Importance/rationale of screening for substance use | Lecture | 30 min |
|  | Learning to screen using ASSIST-Y | Lecture, Group work by peer providers | 1 hour 30 minutes |
|  | Practicing screening using ASSIST-Y | Role plays: Demonstration by facilitators | 1 hour |
|  |  | Role-plays by peer providers | 1 hour 30 minutes |
| **Day 2:** | Practicing to screen using ASSIST-Y | Role-plays by peer providers | 1 hour 30 minutes |
|  | Stages of change | Lecture | 1 hour |
|  | Overview of the screening and brief intervention  Evidence supporting efficacy of screening and brief intervention.  Core components of the ASSIST-linked brief intervention) and motivational interviewing principles and skills (FRAMES model) | Lecture | 2 hours |
|  | Counseling skills: verbal and non-verbal communication, rapport building and self-disclosure, empathy and warmth, normalization of feelings, confidentiality.  A step-by-step approach to delivering the ASSIST-linked brief intervention that incorporates the FRAMES model and motivational interviewing skills | Lecture | 2 hours |
| **Day 3:** | Practicing how to conduct the ASSIST-linked brief intervention; practicing core motivational interviewing skills; Identifying and documenting challenging areas for each peer. | Role plays: Demonstration by facilitators and role plays by peer providers | 4 hours |
|  | Practicing counselling skills; Identifying and documenting challenging areas for each peer. | Role plays: Demonstration by facilitators and role plays by peer providers. | 2 hours 30 minutes |
| **Day 4:** | Practicing challenging areas for each peer provider (identified during previous role-plays) and learning about handling challenging scenarios e.g., youth refusing to see ASSIST-Y scores or not ready to change. | Role plays: Demonstration by facilitators and role plays by peer providers | 3 hours 30 minutes |
|  | Peer provider exam | Role plays: Five standardized scenarios for each peer: (youth with history of no use; youth with moderate risk use and not ready to change; youth with high risk use and ready to change; youth with moderate risk use and undecided about changing; youth who refuses to see ASSIST-Y scores); Rating using a fidelity checklist. | 3 hours |
| **Day 5** | Peer provider exam continued | Role plays: Five standardized scenarios for each peer: (youth with history of no use; youth with moderate risk use and not ready to change; youth with high risk use and ready to change; youth with moderate risk use and undecided about changing; youth who refuses to see ASSIST-Y scores); Rating using a fidelity checklist. | 3 hours |
|  | Peer provider exam continued | Role play exam with one real youth with substance use problems; Rating using a fidelity checklist. | 1 hour 30 minutes |
|  | Post-test quiz | Twenty Multiple Choice Questions | 30 min |
|  | Peer-provider feedback on the Training | Group discussion | 1 hour 30 minutes |
|  |  | **Total Training Time** | **32 hours 30 minutes** |
