# Supplementary material for: Feasibility and acceptability of a peer provider delivered substance use screening and brief intervention program for youth in Kenya
Source: BMC Public Health. 2023 Nov 16;23:2254. doi: 10.1186/s12889-023-17146-w (PMC10652467; doi:10.1186/s12889-023-17146-w)
Supplement: Supplementary file 2 — Additional file 2: Supplementary file 2. Composition of the focus group discussions. [file 12889_2023_17146_MOESM2_ESM.docx]

**Supplementary file 2: Composition of the focus group discussions**

| **FGD** | **Number of participants** | **Date conducted** | **Substance use risk** | **Gender** | **Age** | **Time taken** |
| --- | --- | --- | --- | --- | --- | --- |
| 1 | 5 | 8/03/2022 | moderate and high risk | Male | 19-24 years | 1 hr. 2min |
| 2 | 6 | 10/03/2022 | moderate and high risk | Female | 19-24 years | 1 hr. 36min |
| 3 | 4 | 11/03/2022 | moderate and high risk | Male | 19-24Years | 1 hr. 34 min |
| 4 | 7 | 15/03/2022 | Not used | Female and Male | 15-24 years | 1 hr. 8 min |
| 5 | 3 | 31/3/2022 | moderate and high risk | Male | 15-18 years | 1 hr. 32 min |
|  |  |  |  |  |  | Mean time taken = 1 hr. 22 min |
