# Supplementary material for: Feasibility and acceptability of a peer provider delivered substance use screening and brief intervention program for youth in Kenya
Source: BMC Public Health. 2023 Nov 16;23:2254. doi: 10.1186/s12889-023-17146-w (PMC10652467; doi:10.1186/s12889-023-17146-w)
Supplement: Supplementary file 3 — Additional file 3: Supplementary file 3. Semi structured interview guides for peer providers and clinic leaders. [file 12889_2023_17146_MOESM3_ESM.docx]

**Supplementary file 3: Semi structured interview guides for peer providers and clinic leaders**

**Semi-structured interview guide peer providers**

**Title:** **Feasibility and acceptability of a peer-delivered substance use screening and brief intervention for youth attending Rafiki clinic in Eldoret, Kenya**

**Cover Sheet**

IDI interviewer: ____________________________________________

Date: ____/____/___________(Date/Month/Year)

Start time: ______________________________________________

End time:……………………………………………………..

Face to face/virtual:  ____________________________________

Audio Recordings File: ___________________________

| *Checklist* | | |
| --- | --- | --- |
| **ITEM** | **Y/N** | ***Notes*** |
| Focus group Guide |  |  |
| Recorder (charged, free memory) |  |  |
| Pen |  |  |
| Clipboard |  |  |
| Compensation |  |  |
| Receipt Form |  |  |

**Introduction**

Hi, I am ______ from the Moi Teaching and Referral Hospital/ AMPATH. Thank you for agreeing to take part in this interview.  I am very excited to be here and learn from you.

I am interested in understanding how we can offer substance use counseling for youth and adolescents attending Rafiki clinic.  I wanted to gain your perspective given you are a health care worker at the clinic and an expert at adolescent health.

I have some specific questions I’d like to ask you related to this. Your answers will inform plans to integrate the substance use intervention into routine care at Rafiki.

The research team will also treat this discussion confidentially—which means that we will not use your name or anything else that might identify you in our reports.

Do you have any questions before we begin?

**Participant Information**

- Tell me about your main role at Rafiki and what it entails.
- How long have you served in this role at Rafiki?

**Part 1: Intervention Characteristics**

“Now I’m going to ask you questions about the SBI training:”

1. What was your experience with the SBI training?

What parts of the SBI were easy to learn? Why?

What parts were difficult to learn? Why?

What would you change about the training?

What aspects of the SBI would you need additional training in?

How frequently would you need follow-up trainings?

“Now tell me about you experience offering SBI to the youth at Rafiki.

What parts of the SBI were easy to implement? Why?

What parts of the SBI were difficult to implement? Why?

What challenges/problems did you face when implementing? What went well? (Probe on things like space, noise, privacy, language barrier etc)

1. How useful was the weekly supervision? What did you like about the supervision? What didn’t you like?
2. What would be your recommendations for making SBI work well in Rafiki? (Probe on things like steps in the intervention, cost, staffing, space, follow-up). Do you think Rafiki clinic will be able to make these changes? Why or why not?

**Part 2: Outer Setting**

1. What kind of programs/services are there within the region (outside MTRH) that offer substance use treatment for youth?

- Tell me about those programs?
- How does the Rafiki clinic interact with those programs?
- What works well with those programs?
- What doesn’t work well?

**Part 3: Inner Setting**

1. What are the substance use problems that you see among youth coming to Rafiki? (Probe: Tell me more; give examples)
2. What current resources/services exist at Rafiki to address substance use problems at Rafiki? (Probe: staffing, programs etc)
3. How well does the SBI address the substance use needs of youth at Rafiki?
4. How does SBI fit with the work of a peer at the Rafiki clinic?

**Part 4: Characteristics of Individuals**

1. How confident are you that you will be able to successfully implement the SBI?

What gives you that level of confidence (or lack of confidence)?

**Part 5: Process**

1. What was your role in the planning/implementation of the SBI?
2. Other than Dr. Jaguga or Dr. Apondi, are there people at Rafiki who are likely to champion the SBI? What position do these champions have at Rafiki? How do you think they will help champion for SBI implementation? (Probe: getting people to use the intervention?)
3. How will SBI be made a part of the services offered at Rafiki processes? How will SBI interact or conflict with current programs or processes?
4. What will be required to ensure SBI continues after the grant? (space, funding, staffing, training, places to refer to, support from AMPATH/MTRH)

**Semi-structured interview guide for clinic leaders**

**Title:** **Feasibility and acceptability of a peer-delivered substance use screening and brief intervention for youth attending Rafiki clinic in Eldoret, Kenya**

**Cover Sheet**

IDI interviewer: ____________________________________________

Date: ____/____/___________(Date/Month/Year)

Start time: ______________________________________________

End time:……………………………………………………..

Face to face/virtual:  ____________________________________

Audio Recordings File: ___________________________

| *Checklist* | | |
| --- | --- | --- |
| **ITEM** | **Y/N** | ***Notes*** |
| Focus group Guide |  |  |
| Recorder (charged, free memory) |  |  |
| Pen |  |  |
| Clipboard |  |  |
| Compensation |  |  |
| Receipt Form |  |  |

**Introduction**

Hi, I am ______ from the Moi Teaching and Referral Hospital/ AMPATH. Thank you for agreeing to take part in this interview.  I am very excited to be here and learn from you.

I am interested in understanding how we can integrate substance use counseling for youth and adolescents attending Rafiki clinic.  I wanted to gain your perspective given you are a health care worker at the clinic and an expert at adolescent health.

I have some specific questions I’d like to ask you related to this. Your answers will inform plans to integrate the substance use intervention into routine care at Rafiki.

The research team will also treat this discussion confidentially—which means that we will not use your name or anything else that might identify you in our reports.

Do you have any questions before we begin?

**Participant Information**

- Tell me about your primary role at Rafiki and what it entails.
- How long have you served in this role at Rafiki?

**Outer Setting**

1. What kind of programs/services are you aware of within Eldoret (outside MTRH) that offer substance use treatment for youth?

- Tell me about their programs?
- How does the Rafiki clinic interact with those programs if at all they do?
- What works well with those programs?
- What doesn’t work well?

1. What kind of government policies/guidelines exist that support substance use interventions for youth in Kenya?
2. What kind of government policies/guidelines exist that support substance use interventions for youth in UG county?

**Inner Setting**

1. What are the substance use problems that you see among youth coming to Rafiki? (Probe: Tell me more; give examples)
2. What current resources/services exist at Rafiki to address those needs? (Probe: staffing, programs etc)
3. How well does the SBI address the substance use needs of youth at Rafiki?
4. What resources are available at Rafiki to support SBI implementation? (space, funding, staffing, training, places to refer to)
5. What resources are lacking? What can be done to address the gaps in resources?
6. What kind of MTRH or Rafiki policies exist that support implementation of SBI at Rafiki?
7. How does SBI fit with the overall goals/mission/vision of the Rafiki clinic?
8. How open is Rafiki to new ideas or programs? Tell me an example of a recently introduced program? What made it work? What were the challenges?
9. How high a priority is SBI for Rafiki? Why? What other priorities are higher?

**Characteristics of Individuals**

1. How confident are you that Rafiki Clinic will be able to successfully implement the SBI?

What gives you that level of confidence (or lack of confidence)?

**Process**

“Were you involved in the planning/implementation of the SBI. What was your role in the planning/implementation of the SBI? What went well with the implementation of SBI? What did not go well? What changes can be made?

1. Other than Dr. Jaguga or Dr. Apondi, are there people at Rafiki who are likely to champion the SBI? What position do these champions have at Rafiki? How do you think they will help champion for SBI implementation? (Probe: getting people to use the intervention?; advocating for funding to support the SBI)
2. How will SBI be integrated into current Rafiki processes? How will it interact or conflict with current programs or processes?
3. What will be required to ensure SBI continues after the grant? (space, funding, staffing, training, places to refer to, support from AMPATH/MTRH)
