# Supplementary material for: Feasibility and acceptability of a peer provider delivered substance use screening and brief intervention program for youth in Kenya
Source: BMC Public Health. 2023 Nov 16;23:2254. doi: 10.1186/s12889-023-17146-w (PMC10652467; doi:10.1186/s12889-023-17146-w)
Supplement: Supplementary file 4 — Additional file 4: Supplementary file 4. Additional data on youth substance use characteristics. [file 12889_2023_17146_MOESM4_ESM.docx]

**Supplementary file 4: Additional data on youth substance use characteristics**

**Youth substance use per ASSIST risk levels (n=100)**

| **Substance** | **Risk level/cut-off scores** | **Frequency (%)** |
| --- | --- | --- |
| Tobacco | Moderate (2-11) | 3 (3.0%) |
|  | High (12+) | 2 (2.0%) |
|  | ASSIST score <2 | 95 (95.0%) |
| Alcohol | Moderate (5-17) | 29 (29.0%) |
|  | High (18+) | 6 (6.0%) |
|  | ASSIST score <5* | 65 (65.0%) |
| Cannabis | Moderate (2-11) | 3 (3.0%) |
|  | High (12+) | 7 (7.0%) |
|  | ASSIST score <2 | 90 (90.0%) |
| Cocaine | Moderate (2-6) | 1 (1.0%) |
|  | High (7+) | 1 (1.0%) |
|  | ASSIST score <2 | 98 (98.0%) |
| Khat | Moderate (2-8) | 2 (2.0%) |
|  | High (9+) | 7 (7.0%) |
|  | ASSIST score <2 | 91 (91.0%) |
| Inhalants | Moderate (2-8) | 0 (0.0%) |
|  | High (9+) | 0 (0.0%) |
|  | ASSIST score <2 | 100 (100.0%) |
| sedatives | Moderate (2-6) | 0 (0.0%) |
|  | High (7+) | 1 (1.0%) |
|  | ASSIST score <2 | 99 (99.0%) |
| hallucinogens | Moderate (2-8) | 0 (0.0%) |
|  | High (9+) | 1 (1.0%) |
|  | ASSIST score <2 | 99 (99.0%) |
| opioids | Moderate (2-6) | 0 (0.0%) |
|  | High (7+) | 0 (0.0%) |
|  | Not used | 100 (100.0%) |
| others | Moderate (2-6) | 0 (0.0%) |
|  | High (7+) | 0 (0.0%) |
|  | ASSIST score <2 | 100 (100.0%) |

*Of the 65 who had a score of <5, 6 youth had used alcohol in the past 3 months

**Pattern of poly-substance use among youth with moderate and high-risk substance use (n=100)**

|  | **Frequency (%)** |
| --- | --- |
| Poly-substance use* | 16 (16.0) |
| Alcohol and cannabis | 3 (3.0) |
| Alcohol and khat | 5 (5.0) |
| Alcohol and cocaine | 1 (1.0) |
| Alcohol and tobacco | 1 (1.0) |
| Alcohol sedatives | 1 (1.0) |
| Alcohol, tobacco, cannabis, khat | 1 (1.0) |
| Alcohol, cannabis, and khat | 2 (2.0) |
| Alcohol, tobacco, and cannabis | 1 (1.0) |
| Alcohol, tobacco, and khat | 1 (1.0) |

* Poly-substance use denotes use of more than one substance
